# Synaptic Conductances during Interictal Discharges in Pyramidal Neurons of Rat Entorhinal Cortex

**DOI:** 10.3389/fncel.2016.00233

**Published:** 2016-10-13

**Authors:** Dmitry V. Amakhin, Julia L. Ergina, Anton V. Chizhov, Aleksey V. Zaitsev

**Affiliations:** ^1^Laboratory of Molecular Mechanisms of Neural Interactions, Sechenov Institute of Evolutionary Physiology and Biochemistry of the Russian Academy of SciencesSaint Petersburg, Russia; ^2^Computational Physics Laboratory, Division of Plasma Physics, Atomic Physics and Astrophysics, Ioffe InstituteSaint Petersburg, Russia

**Keywords:** temporal lobe epilepsy, cortico-hippocampal slices, synaptic conductance, NMDA, GABA, AMPA, interictal discharge

## Abstract

In epilepsy, the balance of excitation and inhibition underlying the basis of neural network activity shifts, resulting in neuronal network hyperexcitability and recurrent seizure-associated discharges. Mechanisms involved in ictal and interictal events are not fully understood, in particular, because of controversial data regarding the dynamics of excitatory and inhibitory synaptic conductances. In the present study, we estimated AMPAR-, NMDAR-, and GABA_A_ R-mediated conductances during two distinct types of interictal discharge (IID) in pyramidal neurons of rat entorhinal cortex in cortico-hippocampal slices. Repetitively emerging seizure-like events and IIDs were recorded in high extracellular potassium, 4-aminopyridine, and reduced magnesium-containing solution. An original procedure for estimating synaptic conductance during IIDs was based on the differences among the current-voltage characteristics of the synaptic components. The synaptic conductance dynamics obtained revealed that the first type of IID is determined by activity of GABA_A_ R channels with depolarized reversal potential. The second type of IID is determined by the interplay between excitation and inhibition, with early AMPAR and prolonged depolarized GABA_A_ R and NMDAR-mediated components. The study then validated the contribution of these components to IIDs by intracellular pharmacological isolation. These data provide new insights into the mechanisms of seizures generation, development, and cessation.

## Introduction

A great many *in vitro* and *in vivo* studies have focused on revealing the mechanisms underlying seizures and epilepsy and their treatments, but seizures in many patients with mesial temporal lobe epilepsy remain poorly controlled by antiepileptic drugs (Téllez-Zenteno and Hernández-Ronquillo, 2012). Hence, understanding the pathophysiogenesis of temporal lobe epilepsy remains an important challenge in epilepsy research (Avoli et al., 2002; Löscher, 2011; Curia et al., 2014). Historically, seizures are thought to initiate from decreased inhibition and increased excitation and to terminate when the balance between excitation and inhibition has been recovered. However, a growing body of experimental and theoretical evidence suggests that this is too simplistic a view of the problem (Timofeev and Steriade, 2004).

Seizure synchronization patterns are complex and appear to be related to precise excitatory and inhibitory interactions specific to cell subtypes that occur during seizure initiation, ictal discharge, and termination stages (Ziburkus et al., 2006; Fujiwara-Tsukamoto et al., 2010; Huberfeld et al., 2011; Žiburkus et al., 2013; Avoli et al., 2016). To understand the mechanisms of seizure generation and termination, it is important to know the dynamics of the balance between excitation and inhibition and the changes in synaptic conductance during various stages of epileptiform discharge. These mechanisms are easier to investigate in *in vitro* studies. Of special interest are patterns of epileptiform activity in combined hippocampus-entorhinal cortex slices that resemble limbic seizures in patients with temporal lobe epilepsy (Avoli et al., 2002). Physiological relevance of the *in vitro* models of epileptiform activity have been validated by comparison with experiments in human tissue from patients (Huberfeld et al., 2011; Avoli et al., 2016; de Curtis and Avoli, 2016). However, due to methodological difficulties, the problem of measuring synaptic conductances in a neuron during epileptiform activity, even in *in vitro* models, has been only partially resolved, and data are limited about synaptic conductance dynamics during various types of epileptiform activities (de la Prida et al., 2006; Huberfeld et al., 2011; Žiburkus et al., 2013).

The basic method of estimating synaptic conductance (Borg-Graham et al., 1998; Anderson et al., 2000; Monier et al., 2008; Žiburkus et al., 2013) implies intracellular measurement of synaptic responses at various levels of membrane potential and estimating conductance based on the slope of the obtained current-voltage (I-V) curves. This distinguishes only two types of synaptic inputs, excitatory and inhibitory, and assumes linear dependence of the synaptic currents on membrane voltage. In contrast to evoked responses, epileptiform activity in hyperexcitable epileptic tissue occurs spontaneously and has a different duration and shape, which is why obtaining the precise I-V curves in these experiments is methodologically difficult. Since the excitatory conductance consists of AMPAR- and NMDAR-mediated conductances and the I-V curve of NMDAR-mediated currents is non-linear (Jahr and Stevens, 1990), estimating conductances with an assumption about the linearity of I-V relationships for excitatory currents might cause errors.

In the present study, we estimated AMPAR-, GABA_A_R-, and NMDAR-mediated synaptic conductances during two distinct types of interictal discharges (IIDs) in rat cortical pyramidal neurons of the combined hippocampus–entorhinal cortex slices using dual whole-cell current- and/or voltage-clamp recordings. These conductances were larger than corresponding excitatory and inhibitory conductances calculated based on an assumption about the linearity of I-V relationships for currents. The obtained curves for AMPAR-, GABA_A_R- and NMDAR-mediated synaptic conductances during IIDs may aid further experimental and modeling studies.

## Materials and methods

### Animals

The experiments were carried out in Wistar rats, 20–22 days old. All animal procedures followed the guidelines of the European Community Council Directive 86/609/EEC and were approved by the Animal Care and Use Committee of the Sechenov Institute of Evolutionary Physiology and Biochemistry of the Russian Academy of Sciences.

### Slice preparation

Methods for brain slice preparation were as described previously (Kryukov et al., 2016; Malkin et al., 2016). Rats were sacrificed by decapitation and their brains removed rapidly. A vibrating microtome (Microm HM 650 V; Microm; Germany) was used to cut horizontal slices 300-μm thick that contained entorhinal cortex and hippocampus. All steps used artificial cerebrospinal fluid (ACSF) with the following composition (in mM): 126 NaCl, 2.5 KCl, 1.25 NaH_2_PO_4_, 1 MgSO_4_, 2 CaCl_2_, 24 NaHCO_3_, and 10 dextrose. The ACSF was aerated with carbogen (95% O_2_/5% CO_2_).

### Electrophysiology

Recordings were made at 30°C. Pyramidal neurons in deep layers of the entorhinal cortex were visualized using a Zeiss Axioscop 2 microscope (Zeiss; Oberkochen, Germany) equipped with differential interference contrast optics and a video camera (WAT-127LH; Watec Inc.; Newburgh, NY, USA). Patch electrodes (3–5 MΩ) were pulled from borosilicate-filamented glass capillaries (WPI; UK) on a P-1000 Micropipette Puller (Sutter Instrument; Novato, CA, USA). For current-clamp recordings, a potassium-gluconate-based filling solution (*i*S-1) was used that had the following composition (in mM): 135 K-gluconate, 10 NaCl, 5 EGTA, 10 HEPES, 4 ATP-Mg, and 0.3 GTP (with pH adjusted to 7.25 with KOH). For voltage-clamp recordings, a solution based on cesium-methane-sulfonate (CsMeS) (*i*S-2) was used that had the following composition (in mM): 127 CsMeS, 10 NaCl, 5 EGTA, 10 HEPES, 6 QX314, 4 ATP-Mg, and 0.3 GTP (with pH adjusted to 7.25 with CsOH). For voltage-clamp recordings to block GABA_A_R- (Nelson et al., 1994; Khalilov et al., 2014) and NMDAR-mediated conductances (Berretta and Jones, 1996; Bender et al., 2006) a cesium-fluoride-based solution with MK-801 (*i*S-3) was used that had the following composition (in mM): 131 CsF, 10 NaCl, 5 EGTA, 10 HEPES, 6 QX314, and 3 MK-801 (with pH adjusted to 7.25 with CsOH).

Whole-cell recordings were performed with two Model 2400 (AM-Systems; Sequim, WA, USA) patch-clamp amplifiers, and an NI USB-6211 A/D converter (National Instruments; Austin, TX, USA) using WinWCP5 software (SIPBS; Glasgow, UK). The data were filtered at 10 kHz and sampled at 20 kHz. After formation of the whole-cell configuration, access resistance was <15 MΩ and remained stable (≤30% increase) during the experiments in all cells included.

In the rat combined entorhinal cortex-hippocampal slices, epileptiform activity was induced by epileptogenic low-magnesium solutions with Kv1 channel blocker 4-aminopyridine (4-AP). The first solution (*e*S-1) contained the following (in mM): 125 NaCl, 3.5 KCl, 1.25 NaH_2_PO_4_, 0.25 MgSO_4_, 2 CaCl_2_, 24 NaHCO_3_, 10 dextrose, and 0.1 4-AP. The second solution (*e*S-2) had a high potassium concentration and contained the following (in mM): 120 NaCl, 8.5 KCl, 1.25 NaH_2_PO_4_, 0.25 MgSO_4_, 2 CaCl_2_, 24 NaHCO_3_, 10 dextrose, and 0.05 4-AP. The *e*S-1 solution induced multiple ictal discharges (seizure-like events, SLEs) in the slices, but *e*S-2 induced more IIDs. Both solutions were aerated with carbogen (95% O_2_/5% CO_2_) throughout the experiment. The flow rate in the perfusion chamber was 5–6 ml/min. The liquid junction potentials were measured as described (Neher, 1992), and the holding potential was compensated for offline for voltage-clamp recordings by subtracting 7 mV for *i*S-2 and 5 mV for *i*S-3.

### I-V relationships for AMPAR-, GABA_A_R- and NMDAR-mediated synaptic currents

The I-V relationships for AMPAR-, GABA_A_R-, and NMDAR-mediated synaptic currents were measured from the responses to extracellular stimulation, recorded at various holding potentials from −100 to +40 mV in increments of 5-mV. The stimulating electrode was placed in the same layer of entorhinal cortex as the recorded cell and at a distance of 100–200 μm. Synaptic currents were pharmacologically isolated. AMPAR-mediated synaptic currents were recorded in the presence of AP-5 (50 μM), MK-801 (18 μM), CGP-55845 (5 μM), and bicuculline (20 μM). GABA_A_R-mediated synaptic currents were recorded in the presence of CGP-55845 (5 μM), AP-5 (50 μM), MK-801 (18 μM), and CNQX (20 μM). NMDAR-mediated synaptic currents were recorded in the presence of CNQX (20 μM), CGP-55845 (5 μM), and bicuculline (20 μM). The peak values of these monosynaptic currents were used for the I-V plots. The resulting I-V curves were fitted with various functions using Wolfram Mathematica 10 (Champaign, IL, USA).

The I-V curve of AMPAR-mediated current was fitted with the following linear function:

(1) $$\begin{array}{l} I_{AMPA}\left(U\right)=g_{AMPA}\left(U-V_{AMPA}\right) \end{array}$$

where *V*_*AMPA*_ is the reversal potential of AMPA-mediated current and *g*_*AMPA*_ is the conductance of postsynaptic AMPARs, *U* is the holding potential.

The experimental I-V relationships of GABA_A_R-mediated current was fitted with either a modification of the Goldman-Hodgkin-Katz current equation (Barker and Harrison, 1988):

(2) $$\begin{array}{l} I_{GABA}\left(U\right)=\frac{{\left(zF\right)}^{2}}{RT}PS\;U\frac{\left[C_{out}\right]\left(exp\left(\frac{-zF}{RT}\left(U-V_{GABA}\right)\right)-1\right)}{exp\left(\frac{zFU}{RT}\right)-1} \end{array}$$

or with an exponential function,

(3) $$\begin{array}{l} I_{GABA}\left(U\right)=A\left(exp\left(\frac{\left(U-V_{GABA}\right)}{k}\right)-1\right) \end{array}$$

where *z* is the valence (−1 for chloride); *F*, the Faraday constant (96485 C/Mol); *R*, the gas constant (8.314 J· K^−1^· mol^−1^); *T*, the temperature (303 K); *C*_*out*_, the extracellular chloride concentration (135 mol/m^3^); *V*_*GABA*_, the reversal potential; and *PS* (m^3^/s), the product of membrane permeability for chloride (m/s) and membrane surface area (m^2^). For Equation (2), the varied parameters were *PS* and *V*_*GABA*,_ and for Equation (3), they were *A, k*, and *V*_*GABA*_. To introduce a conductance of GABA_A_R channels, Equations (2) and (3) were rewritten as:

(4) $$\begin{array}{l} I_{GABA}\left(U\right)=g_{GABA}\;f_{GABA}\left(U\right) \end{array}$$

where $g_{GABA}={\frac{dI_{GABA}}{dU}|}_{U\;=\;-20mV}$ is the conductance measured at the membrane voltage level −20 mV, and *f*_*GABA*_(*U*) is the voltage-dependent factor of Equation (2) or (3).

The voltage dependence of the NMDAR currents was approximated using the Bolzman function (Jahr and Stevens, 1990; Harnett et al., 2015):

(5) $$\begin{array}{l} I_{NMDA}\left(U\right)=\frac{g_{inf}}{1\;+\;\exp\left(\frac{V_{12}\;-\;U}{k}\right)}\left(U\;-\;V_{NMDA}\right) \end{array}$$

where *g*_*inf*_ is the receptor conductance without the Mg^2+^ block as *U* approaches infinity; *V*_12_ and *k* determine the voltage dependence of the Mg^2+^ block of NMDARs; and*V*_*AMPA*_ is the reversal potential. *g*_*inf*_, *V*_*NMDA*_, *V*_12_, and *k* were allowed to vary during the approximation. To introduce a conductance of NMDAR channels, Equation (5) was rewritten as:

(6) $$\begin{array}{l} I_{NMDA}\left(U\right)=\;g_{NMDA}\;f_{NMDA}\left(U\right) \end{array}$$

where $g_{NMDA}={\frac{dI_{NMDA}}{dU}|}_{U\;=\;-20mV}$ is the NMDAR conductance measured at the membrane voltage level −20 mV, and *f*_*NMDA*_(*U*) is the voltage-dependent factor of Equation (5).

### Estimation of AMPAR-, GABA_A_R- and NMDAR-mediated conductances

Synaptic conductances during epileptic discharges were estimated from currents recorded at various holding potentials ranging from −100 to +30 mV in increments of 5 mV; 5–25 IIDs at each membrane potential were averaged after subtracting the baseline. I-V curves were formed every 1 ms. AMPAR-, GABA_A_R-, and NMDAR-mediated conductances were estimated by fitting the I-V curves with a 3-parameter function of the total current *I*_*total*_:

(7) $$\begin{array}{r} I_{total}\left(U;g_{AMPA},g_{GABA},g_{NMDA}\right)=g_{GABA}\;f_{GABA}\left(U\right)\\ +\;g_{NMDA}\;f_{NMDA}\left(U\right)\\ +\;g_{AMPA}\left(U-V_{AMPA}\right) \end{array}$$

where *f*_*GABA*_(*U*) and *f*_*NMDA*_(*U*) are the functions from Equations (4) and (6), respectively. Conductances were allowed to vary in a positive range only. The least-square method was utilized to find a good estimate of conductances using Wolfram Mathematica 10.

### Conventional estimation of excitatory and inhibitory conductances

The conventional method of estimating conductances uses a stationary equation for membrane voltage with known reversal potentials and voltage-independent excitatory *g*_*E*_ and inhibitory *g*_*I*_ synaptic conductances (Žiburkus et al., 2013). Thus, the conductances were obtained from fitting I-V relationships with a 2-parameter function at each time point of the IID:

(8) $$\begin{array}{r} I_{total}\left(U;g_{AMPA},g_{GABA},g_{NMDA}\right)=g_{E}\left(U\;-\;V_{E}\right)\;+\;g_{I}\left(U\;-\;V_{I}\right) \end{array}$$

### Statistics

Statistical analysis and plotting were conducted using SigmaPlot 12.5 software (SYSTAT Software; San Jose, CA, USA). Normality of sample data was evaluated with the Kolmogorov-Smirnov test. The Student's *t*-test was used for normally distributed data, and for non-normally distributed data, the Mann-Whitney rank sum test (*U*-test) or the Wilcoxon signed rank test (*W*-test) were used to evaluate statistically significant differences between the values. Differences were considered as statistically significant at *p* = 0.05. The results were expressed as mean ± standard error of the mean.

## Results

We induced epileptiform activity in rat combined entorhinal cortex-hippocampal slices by using two epileptogenic solutions (see Methods). To characterize the synchronized synaptic activity during the main stages of epileptiform activity, we employed dual whole-cell current- and/or voltage-clamp recordings in pyramidal neurons from deep layers of the entorhinal cortex. Synchronized synaptic activity between neighboring pyramidal cells was highly correlated, enabling us to investigate the synaptic currents during various epileptiform discharge patterns.

After perfusing with epileptogenic solutions, we observed increased membrane potential in the pyramidal neurons of from −69 ± 3 to −59 ± 2 mV in *e*S-1 and from the same level to −51 ± 3 mV in *e*S-2. In these neurons, spontaneous depolarizing potentials were observed, and their frequency increased within a few minutes. If *e*S-2 was used, these spontaneous depolarizing potentials sometimes evoked single action potentials. To estimate the changes in spontaneous spiking activity of the excitatory and inhibitory neurons just before epileptiform activity we calculated the frequency of large-amplitude spontaneous synaptic events (>25 pA), which are presumably action potential-evoked responses (Zhang et al., 2014; Gibon et al., 2016). To distinguish the inhibitory and excitatory synaptic events, neurons were voltage-clamped at –27 mV. We found that the frequency of the inhibitory postsynaptic currents (IPSCs) increased from 1.1 ± 0.2 Hz at the beginning of the registration to 5.1 ± 0.2 Hz (*n* = 21, *p* < 0.05, *W*-test) prior to the first synchronized event, while the frequency of excitatory postsynaptic currents (EPSCs) remained the same (0.12 ± 0.04 vs. 0.5 ± 0.2 Hz, *n* = 21, *p* > 0.05, *W*-test).

### Three modes of synchronized synaptic activity

**Figure 1:**
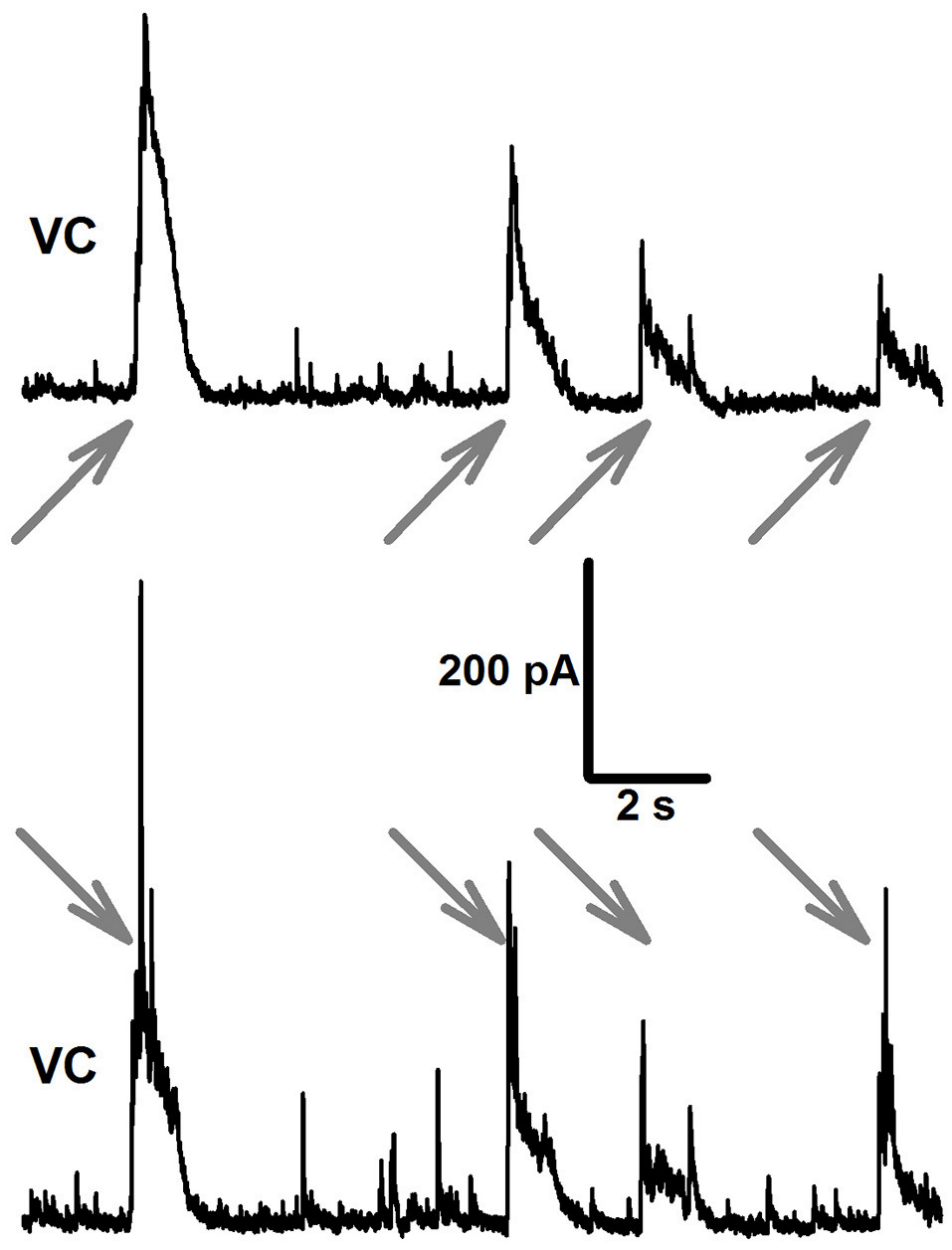
**Mode 1 of synchronized synaptic activity induced by ***e***S-2 in rat entorhinal cortex-hippocampal slices**. Simultaneous voltage-clamp recordings in a pair of neighboring entorhinal pyramidal neurons (*V*_*hold*_ = −27mV, the onset of IID1s is indicated by arrows). Note, that IID1s between pyramidal cells are highly correlated.

The modes of synchronized network activity in slices changed with elapsed time after application of epileptogenic solutions. In 4–6 min after beginning of perfusion of *e*S-1 or *e*S-2, the first synchronized synaptic events were observed. The first mode of synchronized synaptic activity (Mode 1) contained only inhibitory IIDs. Mode 1 was observed in all brain slices and lasted more than 30 min for *e*S-1 but only 4.1 ± 0.8 min (*n* = 17) for *e*S-2. The discharges were composed of high-amplitude bursts of IPSCs that lasted about 1 s each (Figure 1). The frequency of these bursts was higher if *e*S-2 was used (0.022 ± 0.001 Hz for *e*S-1, *n* = 4 and 0.22 ± 0.04 Hz for *e*S-2, *n* = 29, *p* < 0.05, *U*-test). In *e*S-2, the bursts reversed at −54 ± 4 mV (*n* = 5). These bursts will be addressed below as Type 1 IIDs (IID1).

**Figure 2:**
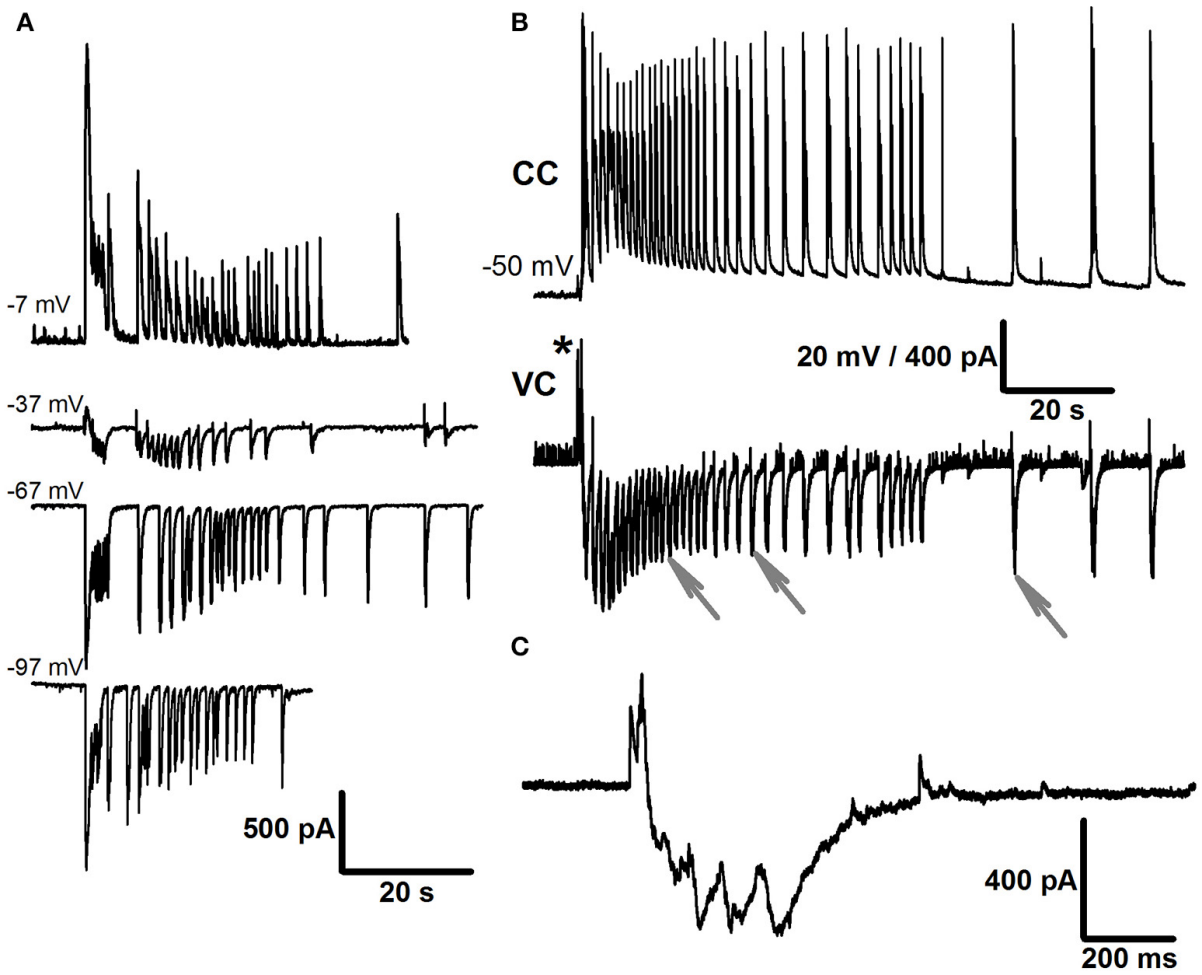
**Mode 2 of synchronized activity**. **(A)** SLEs induced by *e*S-1, recorded in voltage-clamp mode at different holding potentials. Note, that SLE has components that reverse at different voltages. **(B)** SLEs induced by *e*S-2 recorded simultaneously from two neighboring neurons in current clamp (top trace) and voltage clamp (bottom trace, *V*_*hold*_ = −27mV). Asterisk indicate the initial burst of IPSCs at the beginning of SLE. Stereotypical current patterns (arrows) compose the main part of SLE. One of the patterns is extended in **(C)**.

Then, in most slices, the first mode of activity was replaced with the second mode of synchronized activity (Mode 2), which was characterized by the presence of ictal discharges (or seizure-like events, SLEs; Figures 2A,B) interleaving with IID1. SLEs were observed in 100% and 58% of slices with *e*S-1 and *e*S-2, respectively. In current-clamp recordings, this type of activity was characterized by action potential burst discharges lasting for 30–80 s (Figure 2B, top trace). Simultaneous dual whole-cell recordings in current- and voltage-clamp modes revealed that every SLE began with either a short burst of high-amplitude IPSCs [burst amplitudes could reach several nA, and an example of an IPSC burst is marked with asterisks (^*^) in Figure 2B] or a prolonged burst of IPSCs having a lower amplitude. The main part of each SLE was composed of stereotypical bursts of currents with components that reversed at various voltages (Figures 2A,B). This stereotypical current burst lasted about 1 s and had 1–2 fast IPSCs at the beginning, followed by 3–5 overlapping EPSCs (Figure 2C). In most cases, these stereotypical current bursts were clustered at the onset of the SLE, forming the “tonic” phase of the ictal event (**Figure 4A**, upper trace). However, in some cases, these current bursts emerged at lower frequencies and formed “clonic” SLEs like the one in **Figure 4A** (lower trace).

**Figure 3:**
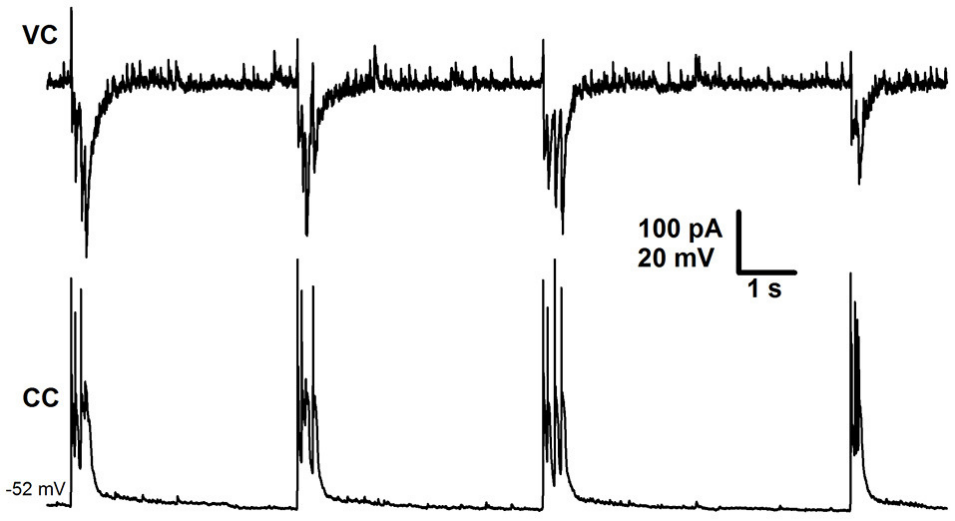
**Mode 3 of synchronized activity induced by ***e***S-2**. Simultaneous voltage- (*V*_*hold*_ = −27mV) and current-clamp recordings from two neighboring cells.

The duration of Mode 2 depended on the type of solution used. When slices were perfused with *e*S-1, this mode could last several hours, resulting in recording of more than 20 SLEs (1–2 SLEs every 5 min). Administering *e*S-2 rarely resulted in more than 5 SLEs. Further, exposing the slice to *e*S-2 induced transition of synchronized synaptic activity to the third mode, which was not observed if *e*S-1 was used. The third mode of synchronized activity (Mode 3) was characterized by the presence of stereotypical current bursts (Figure 3) similar to those observed during SLEs (Figure 2C). In contrast to those in previous modes, these current bursts did not cluster but emerged regularly with low frequency (0.24 ± 0.02 Hz, *n* = 22). We referred to these current bursts as Type 2 IIDs (IID2). This mode of synaptic activity could last several hours. Simultaneous voltage- and current-clamp recordings demonstrated that each IID2 corresponded to bursts of action potentials in pyramidal cells (Figure 3).

**Figure 4:**
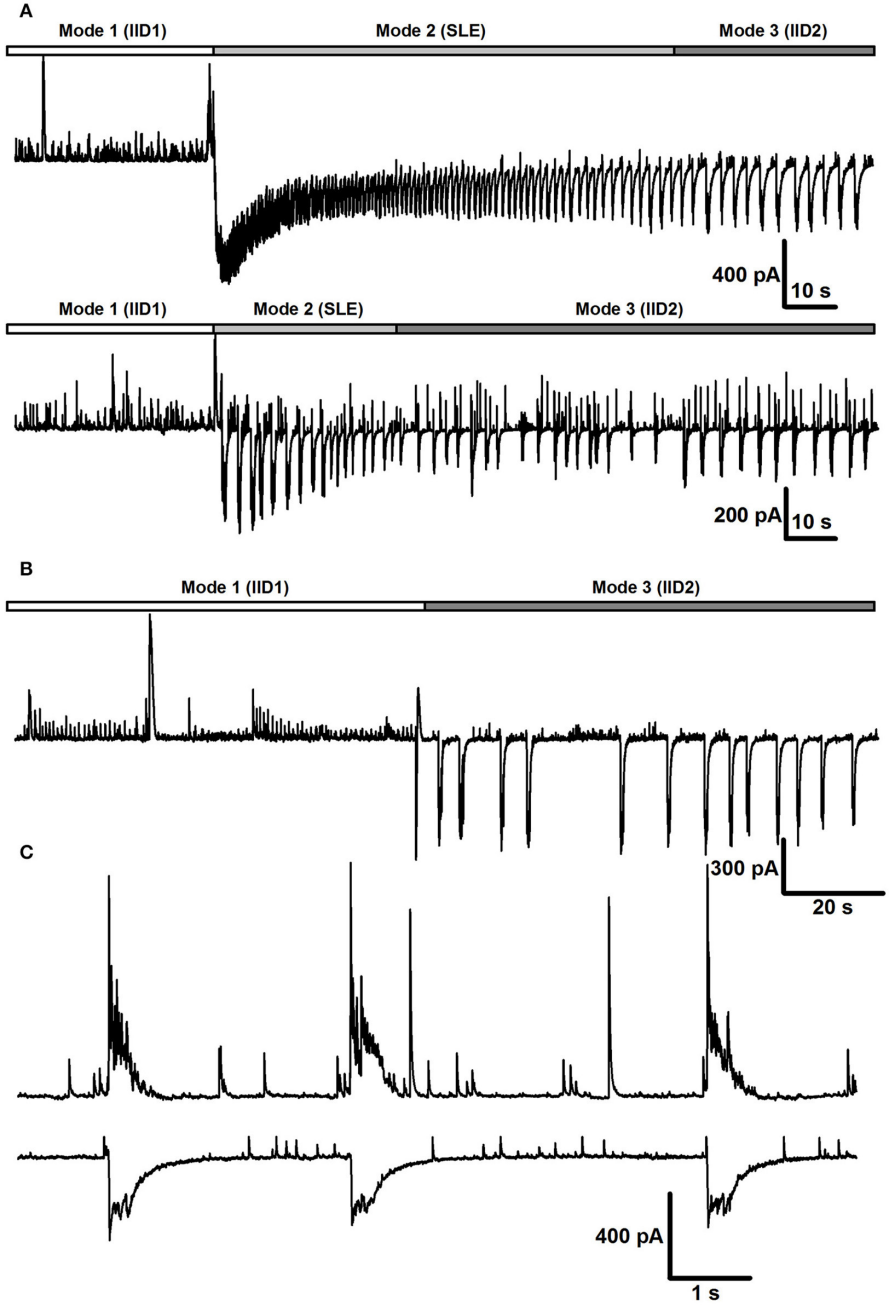
**The transitions between modes of the synchronized synaptic activity**. **(A)** Two representative traces recorded in the voltage-clamp mode in different slices with all three modes. Both tonic and clonic phases of SLE are present in the upper trace, whereas only clonic phase is in the lower trace. **(B)** In some cells, the Mode 1 proceeded to the Mode 3 directly **(C)**. Simultaneous voltage-clamp recordings (*V*_*hold*_ = −27mV) in a pair of cells that receive different types of synaptic inputs during the mode 3. First cell (top trace) displays IID1, the second one displays IID2.

The transitions between modes of the synchronized synaptic activities described were observed in the majority of slices during application of the *e*S-2 and are summarized in Figure 4A. However, in some slices, we did not observe all modes. For example, in 27% of slices, the first mode of activity was the only one, and in other slices (18%), the activity skipped SLEs and proceeded directly to the third mode (Figure 4B). In addition, simultaneous dual whole-cell recordings in voltage-clamp mode showed that in some cases (<5% of slices) two neighboring neurons exhibited differing types of IIDs (Figure 4C). In other words, IID1 and IID2 could coexist in adjacent neurons in the same slice.

### Assessing various synaptic conductances during IID1 and IID2: numeric optimization approach

**Figure 5:**
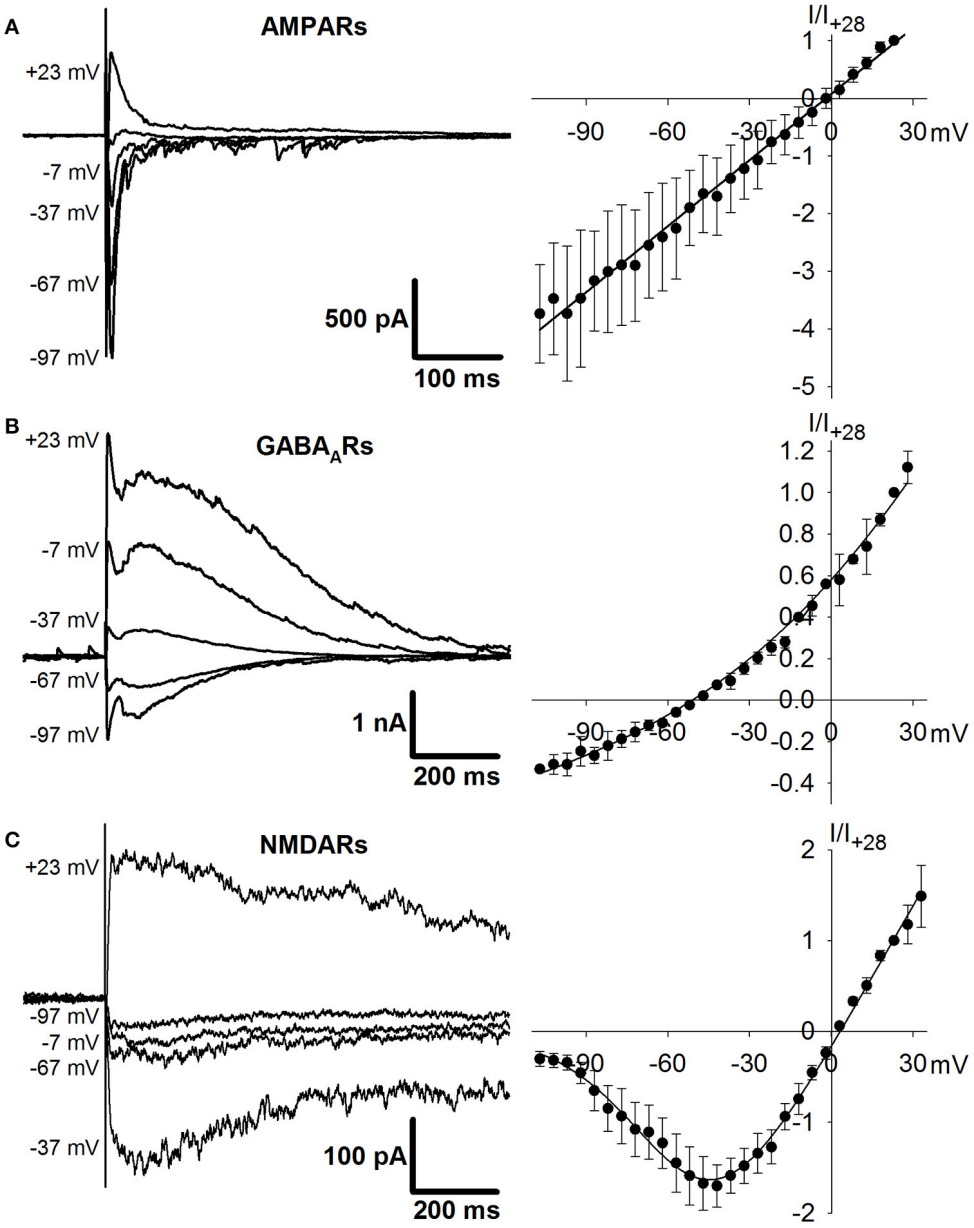
**The I-V relationships of AMPAR (A), GABA_**A**_R (B), and NMDAR-mediated (C) currents**. Representative examples of evoked pharmacologically isolated postsynaptic currents recorded at different holding potentials in *e*S-2 (left panels). Experimentally obtained I-V relationships are plotted in the right panels (circles) and fitted with Equations (1), (2), and (5; solid lines). *n* = 6, 8, and 5 neurons for AMPARs, GABA_A_Rs, and NMDARs, respectively.

**Figure 6:**
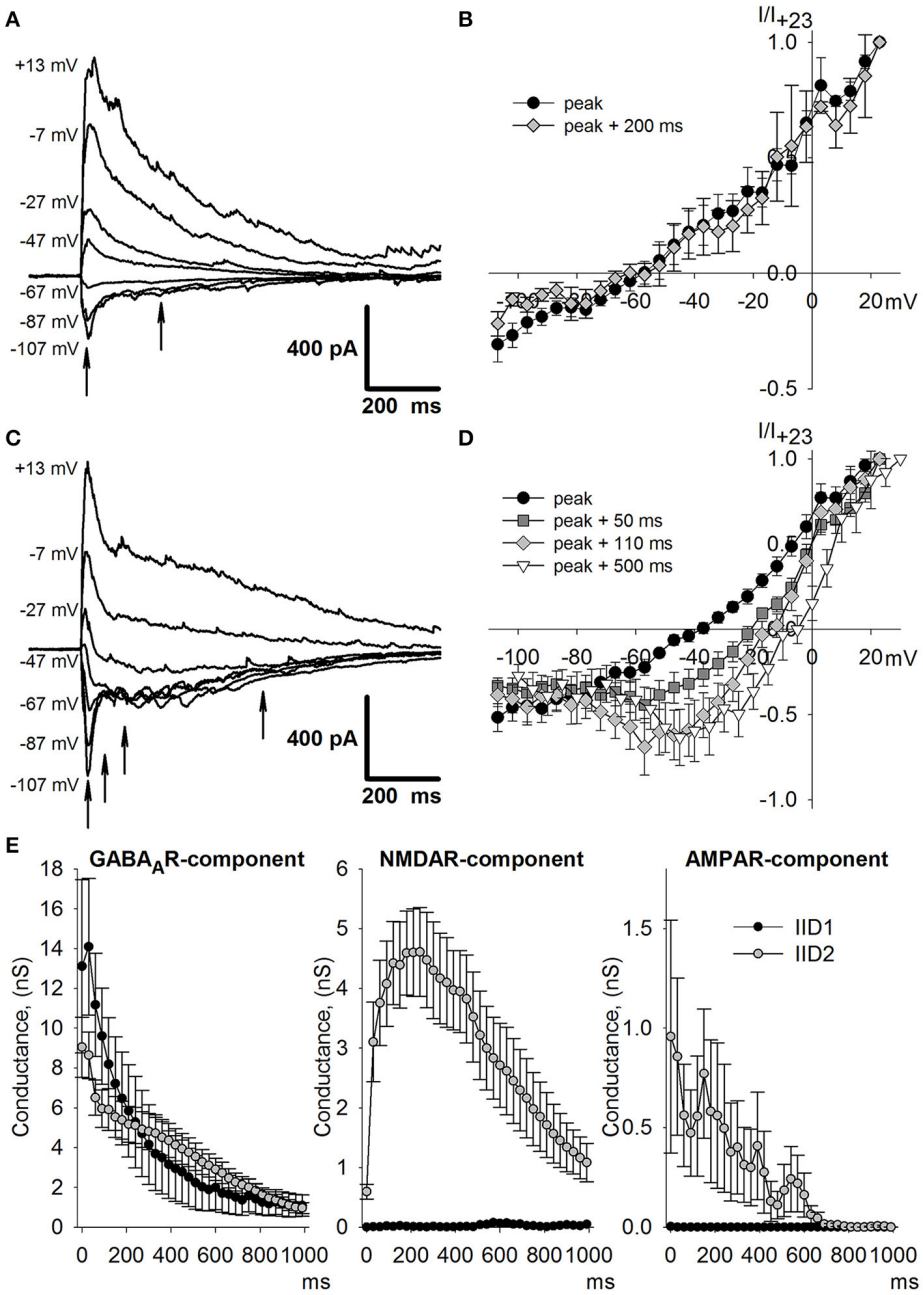
**Assessing synaptic conductances during IID1 and IID2**. Average IID1 **(A)** and IID2 **(C)** voltage-clamp recordings at different membrane potentials. Arrows indicate the time points for which I-V relationships are shown in **(B)** and **(D)**, accordingly. **(E)** The estimated conductances as functions of time for IID1 (black dots) and IID2 (white dots). IID1 is GABA_A_R-mediated, while IID2 is composed of all the synaptic components. *n* = 5 and 10 neurons for IID1 and IID2, respectively.

To investigate which types of postsynaptic receptors underlie IID1 and IID2, we further estimated synaptic conductances by conducting recordings at various levels of membrane potential. To assess the effects of AMPAR-, GABA_A_R-, and NMDAR-mediated synaptic conductances during IID1 and IID2, we used the numerical optimization approach. First, we determined the I-V relationships of AMPAR-, NMDAR-, and GABA_A_R-mediated currents, as described in the Methods Section. Figure 5 shows representative examples of recordings and I-V curves fitted with Equations (1), (2), and (5). Next, we recorded IID1 and IID2 at various holding potentials and averaged 5–25 of them at each membrane potential (Figures 6A,C show IID1 and IID2, respectively). Then, we built a set of I-V curves at various time points from the beginning of IIDs (Figures 6B,D) and fitted them with Equation (7) using the conductances *g*_*AMPA*_, *g*_*GABA*_, and *g*_*NMDA*_ as coefficients. No assumptions on a concomitant activation of different receptors have been made. Figure 6E plots the calculated conductances as functions of time. Our estimation revealed that IID1 was an almost purely GABA_A_R-mediated response, as the contribution of the excitatory postsynaptic conductances was negligible. GABA_A_R-mediated conductance also dominated during the initiation of IID2, but later, NMDAR-mediated conductance prevailed. An unexpected result of our numerical study was the very low impact of AMPAR-mediated conductance during IID2. These results suggest that AMPARs are activated with some delay after GABA_A_R and that their impact peaks at the beginning of the discharge and then declines to be quite low during most of the IID2.

### Validating numeric assessment of synaptic conductance with intracellular blockage

**Figure 7:**
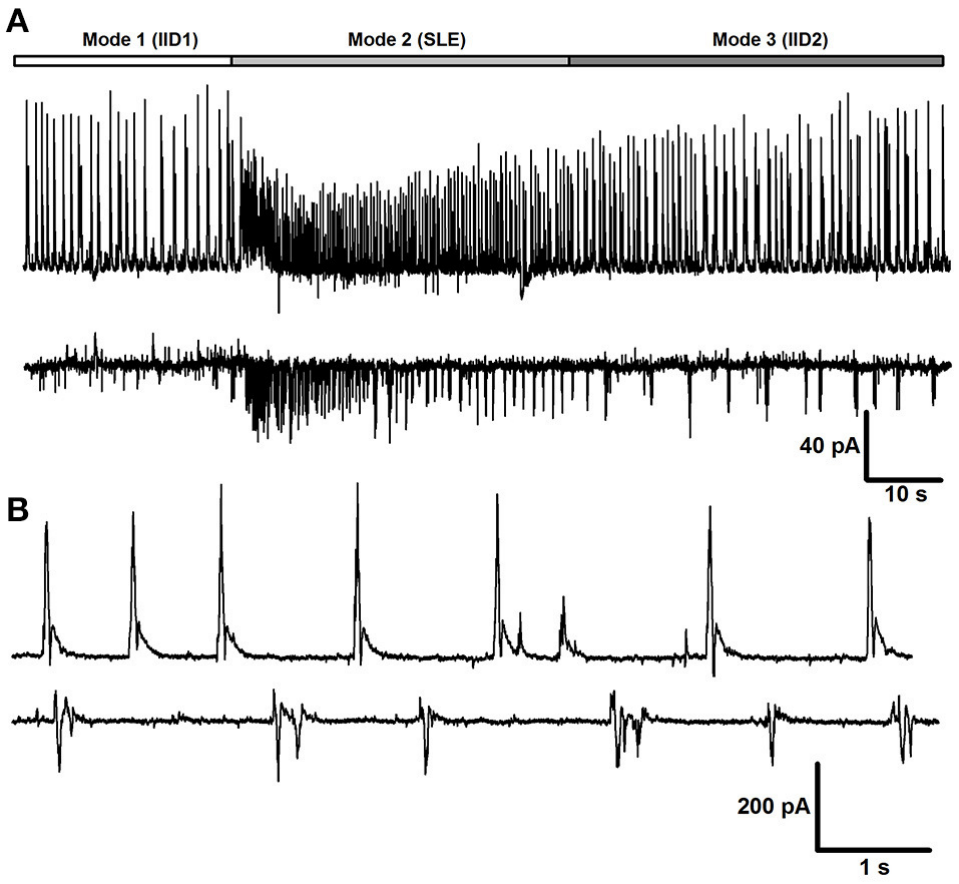
**Synchronized synaptic activity induced by ***e***S-2 and recorded with ***i***S-3 pipette solution, which completely blocks NMDAR-mediated current and partially inhibits GABA_**A**_R-mediated current**. **(A)** Two representative examples of voltage-clamp recordings (*V*_*hold*_ = −25mV) with incomplete (upper trace) and almost full blockage of GABA_A_Rs (lower trace). Note that during all modes of activity in the upper panel the residual positive GABA_A_R-mediated current masks negative AMPAR current; AMPAR current prevails in the lower neuron. **(B)** Expanded records of IID2s from **(A)**. In both neurons, AMPAR-mediated current follows GABA_A_R-mediated current with a delay.

To independently evaluate the involvement of AMPARs during synchronized synaptic activity and validate the numeric optimization approach, we attempted to isolate AMPAR-mediated currents. Since bath application of GABA_A_R or NMDAR antagonists affects epileptiform network activity (Avoli et al., 2002), we blocked GABA_A_Rs and NMDARs from inside the cell by using a fluoride-based internal solution containing 3 mM of the NMDAR blocker MK-801 (*i*S-3). This approach allows to keep synchronized synaptic activity in slice undisturbed. First, we checked whether *i*S-3 blocked NMDAR-mediated conductance. Applying bicuculline (20 μM) and CNQX (20 μM) abolished the postsynaptic currents evoked by extracellular stimulation, indicating that *i*MK-801 completely blocked NMDARs in the recorded neurons. Long-lasting whole-cell recordings with a fluoride-containing intracellular solution lacking ATP and GTP have been reported as blocking GABA_A_R-mediated currents (Khalilov et al., 2014). Using *i*S-3, we found that in the presence of CNQX (20 μM), AP5 (50 μM), and MK-801 (18 μM), the amplitude of evoked IPSCs (eIPSCs) decreased 50–80% over time but the eIPSCs were not fully blocked in some neurons, even after 45 min of whole-cell recording. The remaining response was completely abolished by bicuculline (20 μM), indicating that in our preparation, the intracellular fluoride ions were unable to completely block GABA_A_R-mediated currents, although the *i*S-3 significantly decreased GABA_A_R-mediated conductance. All modes of synchronous synaptic activity described above were observed when *i*S-3 was used (Figure 7).

**Figure 8:**
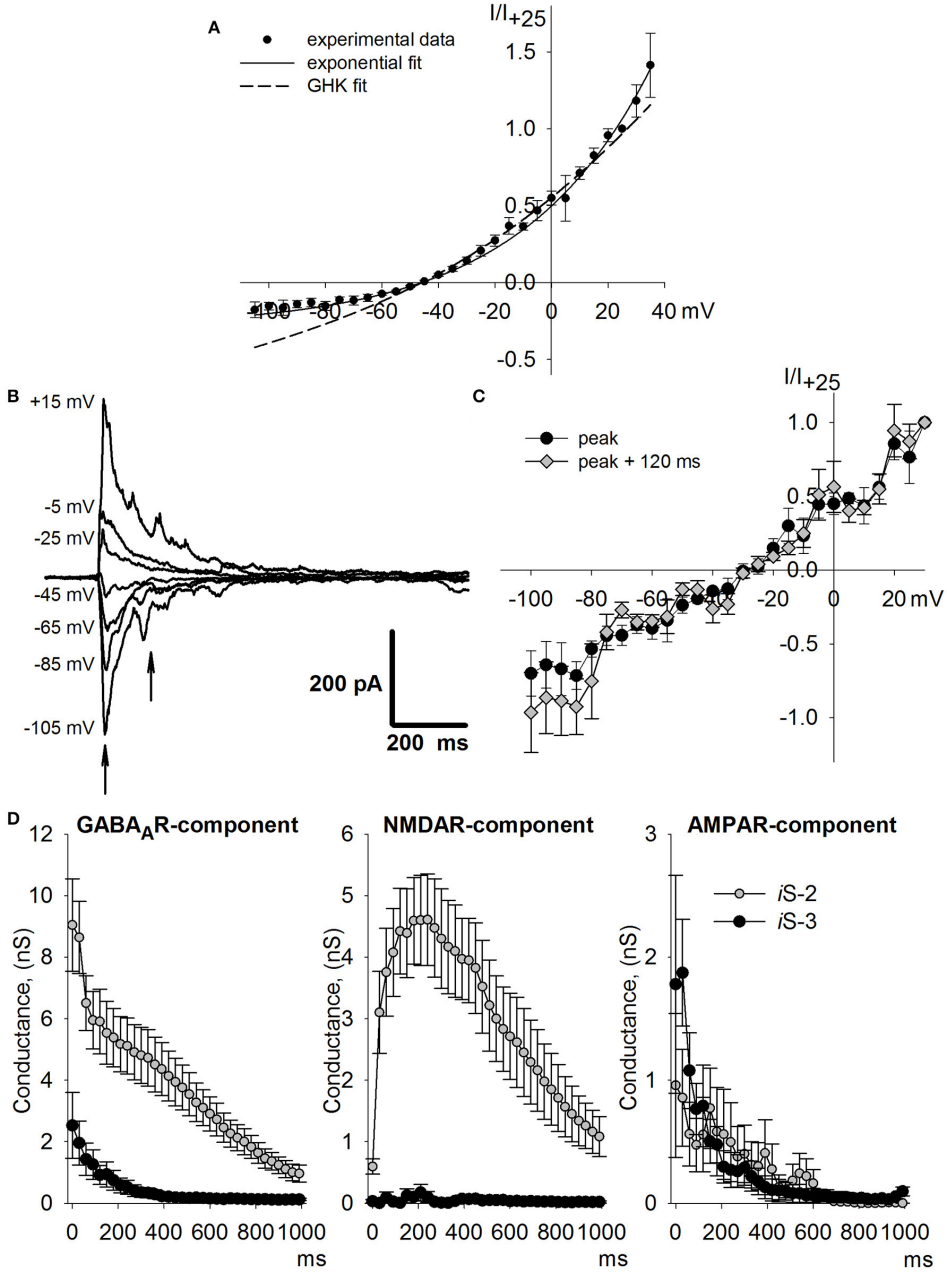
**Assessment of synaptic conductance with intracellular blockage**. **(A)** Experimentally obtained I-V relationship for GABA_A_R-mediated current (dots, *n* = 5 neurons) fitted with Equation (2; dashed line) and Equation (3; solid line). **(B)** Average IID2 voltage-clamp recordings at different membrane potentials. Arrows indicate the time points for which I-V relationships are shown in **(C)**. **(D)** The estimated conductances as functions of time for IID2 recorded with *i*S-3 (black dots) or *i*S-2 (white dots). Intracellular fluoride in *i*S-3 substantially decreases the GABA_A_R-mediated conductance, while *i*MK-801 completely blocks NMDAR-mediated conductance. *i*S-3 does not change AMPAR-mediated peak conductance (*p* > 0.05, *U*-test).

**Figure 9:**
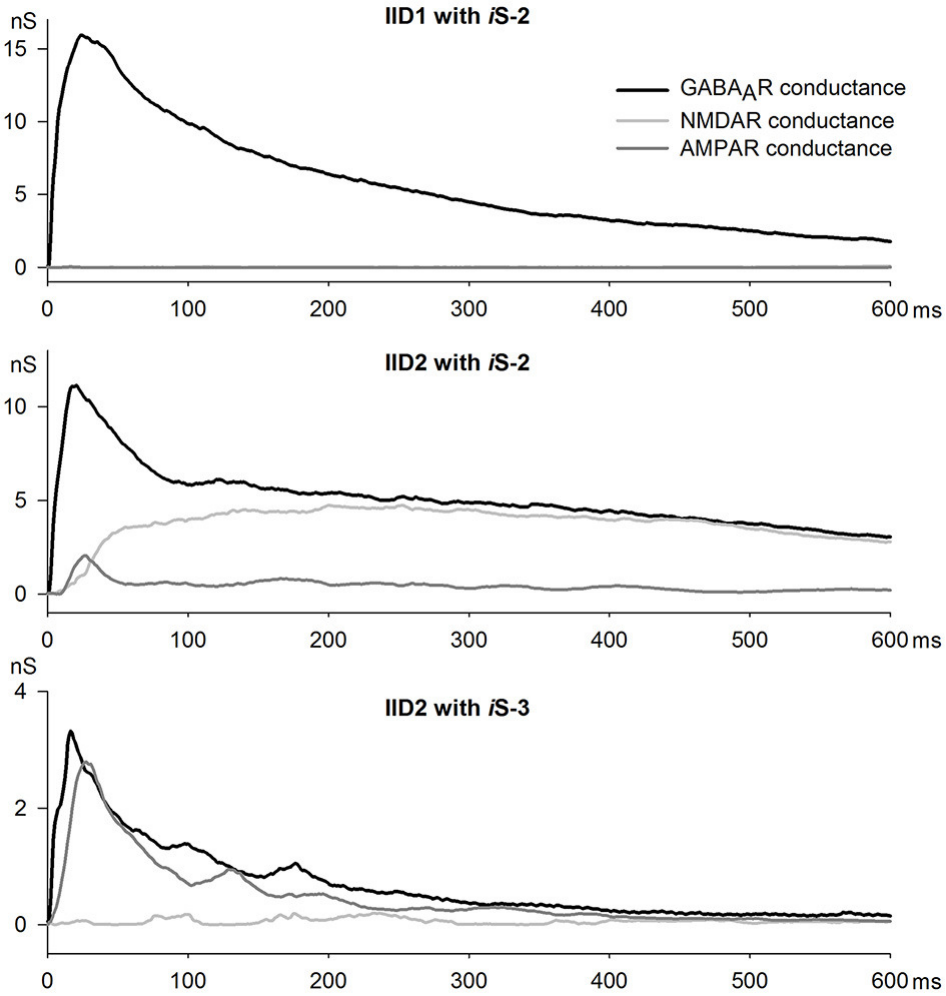
**The dynamics of synaptic conductances during IID1 (top panel) and IID2 (middle and bottom panels) plotted with high temporal resolution (1 ms)**.

Next, we estimated the synaptic conductances during IID2 in this preparation (Figure 8). Because we used various intracellular solutions in these experiments, we determined the I-V relationships of AMPAR-, GABA_A_R-, and NMDAR-mediated currents in this preparation. To enable recording of NMDAR-mediated EPSCs, no MK-801 was added to *i*S-3. No difference was found in either AMPAR or NMDAR I-V relationships compared to the ones obtained with *i*S-2 (data not shown). However, the I-V curve of GABA_A_R-mediated currents differed from that obtained with *i*S-2, and the curve shifted 5 mV to the left and had a stronger outward rectification than predicted by the Goldman-Hodgkin-Katz current equation, Equation (2; Figure 8A vs. Figure 5B). We fitted the obtained I-V curve with Equation (3). The IID2 in this preparation (Figure 8B) did not exhibit the late phases of the discharge (for comparison, see Figure 6C), which presumably depended on activating GABA_A_R- and NMDAR-mediated synaptic conductances. The I-V relationships obtained at various time points during IID 2 (Figure 6D) were nearly linear and had reversal potentials between those of GABA_A_R- and AMPAR-mediated reversals. The calculated conductances (Equation 7) of the synaptic receptors were plotted as a function of time (Figures 8D, 9, lower trace). Due to partial blocking by intracellular fluoride, the *g*_*GABA*_ values obtained were much lower than those obtained with *i*S-2 (Figure 6E). Note, that no contribution of NMDAR conductance was found because the *i*MK-801 completely blocked the NMDARs. Estimates of *g*_*AMPA*_ were very similar to those obtained using *i*S-2. AMPARs were activated with a slight delay after GABARs (Figure 9).

### Temporal separation of synaptic components during IID2

**Figure 10:**
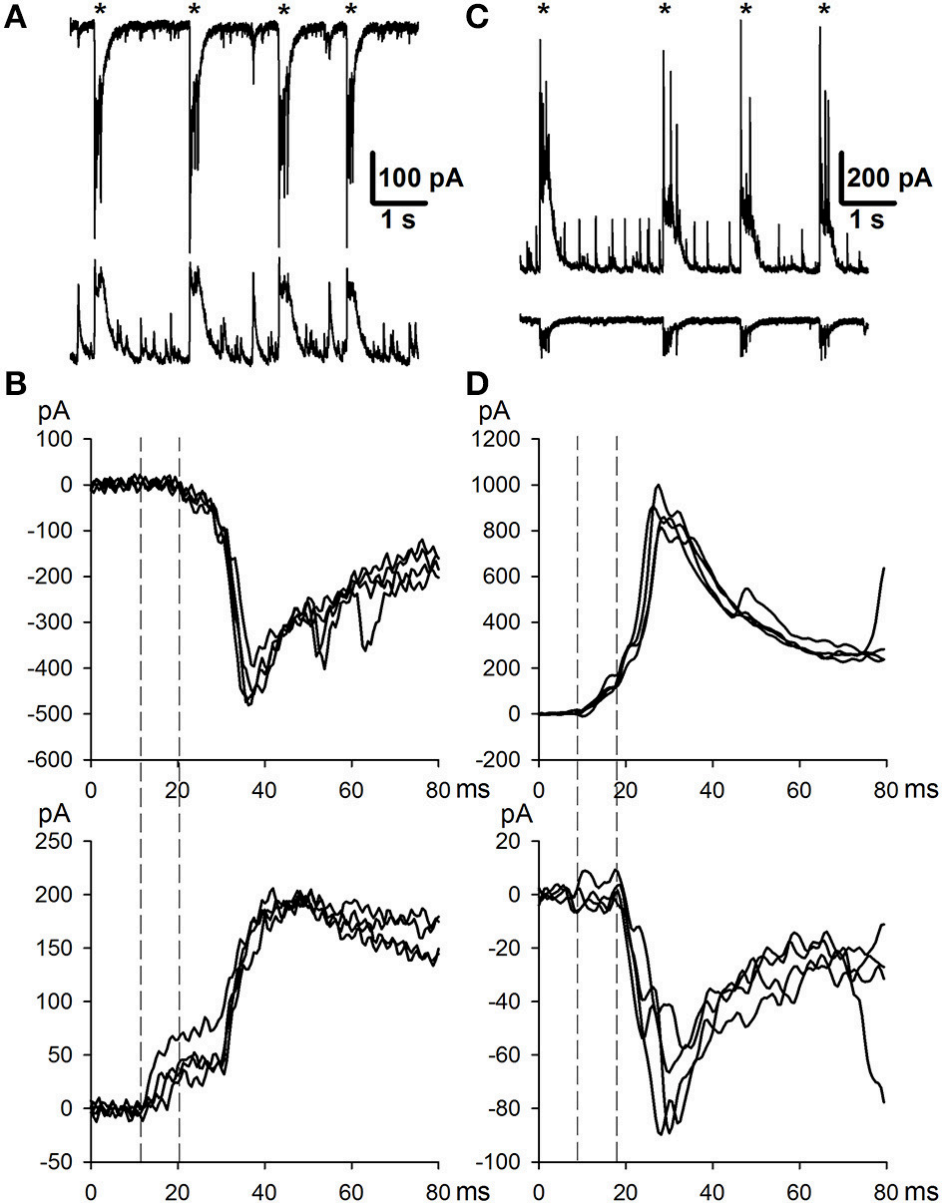
**Temporal separation of inhibitory and excitatory synaptic components during IID2, revealed by dual-cell voltage-clamp recordings**. **(A)** The membrane currents recorded at holding potential equal to the reversal potential of glutamate-mediated currents for one cell (top) and equal to the reversal potential of GABA_A_R-mediated current for the other (bottom). **(B)** Inset demonstrating earlier onset of IID2 for the second cell. **(C,D)** Similar recordings for the same cells with swapped holding voltages. Asterisks indicate IID2s, which are superimposed and expanded in **(B)** and **(D)**.

Obtained estimates of synaptic conductances during IID2 indicate that GABAergic component starts earlier than glutamatergic one. In order to reveal the temporal separation of current onsets, we performed simultaneous voltage-clamp recordings from two neighboring cells. One neuron was clamped at the reversal potential of GABA_A_R-mediated current; the other neuron was clamped at the reversal potential of glutamatergic current (Figure 10). Under these conditions, the temporal separation between the inhibitory and excitatory current onsets was clearly observed (Figure 10B). Moreover, when we switched voltage potentials between cells, the temporal separation of the onsets of the inhibitory and excitatory currents persisted (Figures 10C,D).

### Comparison with conventional method of estimating synaptic conductance

**Figure 11:**
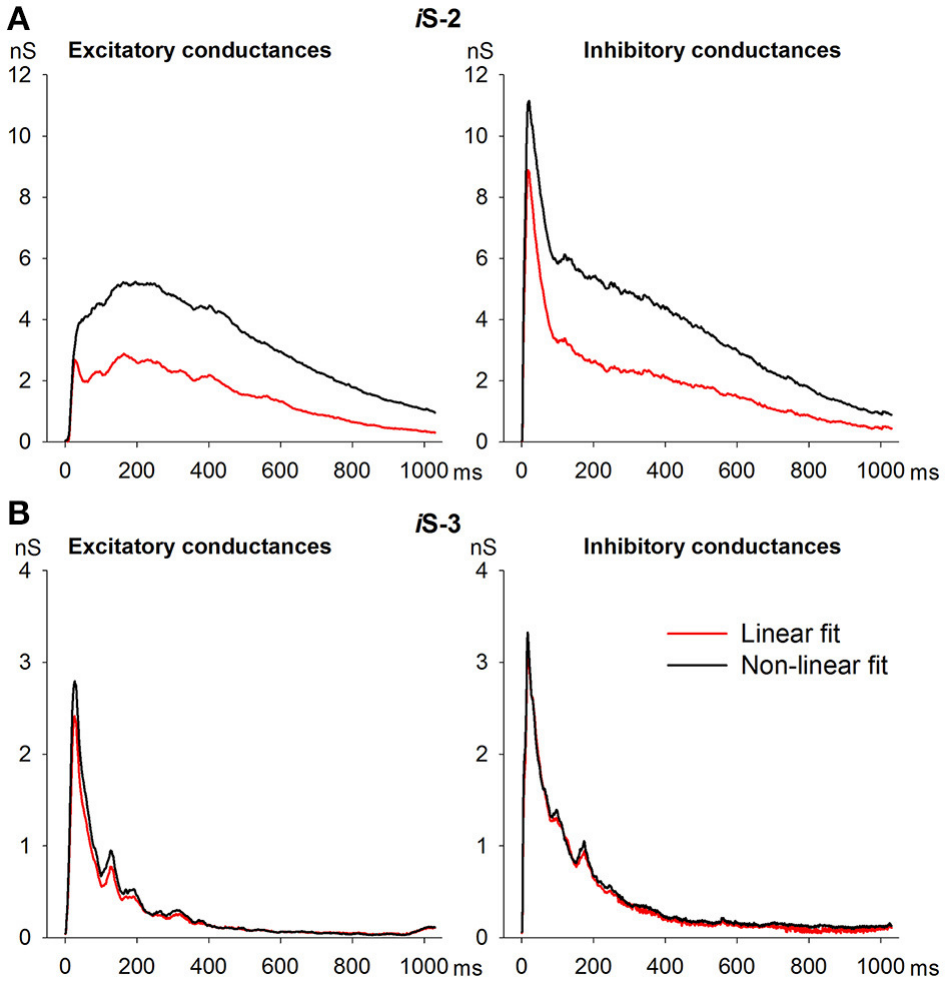
**Comparison of methods of synaptic conductance estimation**. **(A)** An assumption about the linearity of I-V relationships for synaptic currents (red lines) results in underestimation of both excitatory and inhibitory conductances in comparison to those obtained with realistic I-V relationships (black lines). **(B)** In the case of NMDAR blockage, the estimates by the two methods are close.

Previous studies that have estimated synaptic conductances during various forms of synchronized synaptic activity (Shu et al., 2003; Rudolph et al., 2007; Žiburkus et al., 2013) have not subdivided the excitatory conductance (*g*_*E*_) into AMPAR and NMDAR components. To compare the method of estimating conductance used in the present study with those used in previous studies (Žiburkus et al., 2013), we approximated the set of I-V relationships for IID2 by linear function, Equation (8), with *g*_*E*_ and *g*_*I*_ as unknown coefficients. The conductance traces obtained (Figure 11A, red lines) differed from *g*_*AMPA*_ + *g*_*NMDA*_ and *g*_*GABA*_ (Figure 11A, black lines). The estimated *g*_*I*_ during IID2 was lower than *g*_*GABA*_ by almost two-fold (Figure 11A, right), and *g*_*E*_ was also two times lower than the sum of *g*_*AMPA*_ and *g*_*NMDA*_ (Figure 11A, left). Thus, these two methods produce inconsistent results. We suggest that the main source of inconsistency is the conventional approach's assumption of the linearity of the I-V relationships for excitatory currents. Because the effect of *g*_*NMDA*_ is higher than that of *g*_*AMPA*_ during IID2 and the I-V curve of NMDARs is not linear, this assumption should not be made. To clarify this issue we performed estimates of conductances using *i*S-3 which blocks NMDARs. We found that in this preparation, estimates obtained with both methods were similar (Figure 11B), confirming our explanation. Taken together, these data demonstrate that our measurement technique for various synaptic conductances is reasonably accurate. They also suggest that assumption about the linearity of I-V relationships for excitatory currents should be made with care in studies of epileptiform events.

## Discussion

To our knowledge, this is the first study of AMPAR-, GABA_A_R-, and NMDAR-mediated synaptic conductance dynamics in cortical pyramidal neurons during IIDs. Calculated synaptic conductance dynamics showed that IID1 was determined by hyperpolarized GABA_A_R-mediated components. During IID2s, the balance shifted toward the interplay between excitation and inhibition, with early hyperpolarized GABA_A_R-mediated components and prolonged NMDAR-mediated components.

### Methodological issues

Many previous studies have estimates only excitatory and inhibitory synaptic conductances during various types of epileptiform activities (de la Prida et al., 2006; Žiburkus et al., 2013). Because the basic assumptions made are crucial to the results of such estimates, we focused on the details of the methodological approaches and their validation. The proposed method for estimating conductance is a modification of the most-basic one (Borg-Graham et al., 1998; Anderson et al., 2000; Monier et al., 2008), which implies intracellular measurements of membrane voltage or current at various levels of membrane potential and requires repeated recordings. In the case of spontaneous activity, the method is applicable only if the most important data is contained in the signals that are averaged over several trials. The basic method assumes only two types of synaptic input (excitatory and inhibitory), known reversal potentials for both excitatory and inhibitory currents, and synaptic currents linearly dependent on voltage. Algebraic calculations provide estimates for the corresponding conductances if the averaged synaptic responses are recorded at two or more different membrane potentials. In spite of its common use in *in vivo* and *in vitro* studies, this method cannot be extended to measure three or more conductances while assuming the linear-voltage dependence of the synaptic currents, because under such conditions, only two input variables control a neuron (Pokrovskii, 1978; Odom and Borisyuk, 2012; Chizhov et al., 2014). In the present study, we estimated three conductances by exploring the non-linearity of NMDAR-mediated current and using direct measurements of current-voltage dependences for each synaptic component and its reversal potential.

Comparing our estimates with those obtained by the conventional method of estimating synaptic conductance identified underestimation of both excitatory and inhibitory conductances inherent in the conventional approach (Figure 11A). We suggest that this is caused by neglecting the voltage dependence of NMDAR conductance. We verified this cause by comparing the estimates made using both methods in the presence of an NMDA blocker (Figure 11B). In fact, the voltage-dependence of NMDAR-mediated conductance has an amplifying effect that exposes negative conductance (Smirnova et al., 2015), similar to the effect of persistent sodium channels, which is explained in detail by Vervaeke et al. (2006). This effect might be unavoidable in single-trial conductance estimates (Chizhov et al., 2014).

Other potential sources of errors when estimating conductances include the trial-to-trial and event-to-event variations in amplitude and time course; the electrode series resistance contamination (Karlsson et al., 2011); the interplay of various receptors (Bai et al., 2002); the change of reversal potentials during single recorded events (Mathias et al., 1990; Yelhekar et al., 2016), especially the chloride-dependent GABA_A_R reversal potential (Glykys et al., 2014); and the non-stationary effects of voltage-gated ionic channels, caused, in turn, by non-ideal space clamp conditions (Poleg-Polsky and Diamond, 2011).

### Effects of high potassium concentration

Extracellular potassium concentration is a critical factor that determines the mode of synchronized activity (Fröhlich et al., 2010; Raimondo et al., 2015). Our experimental models of epileptiform activity implied a shift of [K^+^]_o_ to 3.5 mM in *e*S-1 and to 8.5 mM in *e*S-2. We found that SLEs were more reliably induced with eS1, than with eS2. It is currently unclear, why the increased [K^+^]_o_ in our preparation partially prevents the emergence of SLEs and provokes IID2s with relatively low frequency (0.24 ± 0.02 Hz).

It was reported previously that synchronous activity of neurons and high activation of GABA_A_Rs increases the extracellular potassium concentration up to 10–14 mM during SLEs (Avoli and de Curtis, 2011). Potassium concentration tops at the tonic phase of SLE and slowly decreases during the clonic phase (Raimondo et al., 2015). Increasing [K^+^]_o_ has multiple effects. Neurons and glial cells become more depolarized when [K^+^]_o_ increases (Antonio et al., 2016). Changes in extracellular potassium may suffice to induce seizure-like firing (Bazhenov et al., 2004; Ullah et al., 2009; Krishnan and Bazhenov, 2011). High [K^+^]_o_ reduces the driving force for K^+^ currents through Kv channels, thereby prolonging the duration of action potential and consequently increasing transmitter releases, including GABA and glutamate. This results in a large increase in membrane conductance in the cortex and hippocampus (Syková, 1983).

The transmembrane K^+^ gradient provides the driving force for potassium-chloride cotransporter 2 (KCC2), which mediates Cl^−^ extrusion in the neurons. Hence, high [K^+^]_o_ interferes with Cl^−^ transport (Liotta et al., 2011), leading to a depolarizing shift in Cl^−^ equilibrium potential, which, in turn, reduces the efficacy of inhibition (Thompson and Gähwiler, 1989; Lopantsev et al., 2009; Doyon et al., 2016). Indeed, we found that when [K^+^]_o_ was elevated, the GABA equilibrium potential shifted to membrane-potential values that were more depolarized (Figure 5).

### Types of epileptiform discharges

Various types of epileptiform synchronization can be observed in combined slices of the hippocampus-entorhinal cortex when these slices are maintained *in vitro* and continuously perfused with ACSF containing convulsant drugs and/or low [Mg^2+^]_o_ (Avoli et al., 2002). In our preparation, the GABA_A_R-mediated IID1 properties resembled those of a previously described events referred to as slow interictal potentials (Avoli et al., 2002). At resting membrane potentials, these GABA_A_R-mediated, slow interictal events are characterized by a long-lasting depolarization that is accompanied by minimal action potential firing (Avoli et al., 2002). The slow interictal events generated by parahippocampal networks can vary among experiments, both in their occurrence interval (between 2.5 and 50 s) and in their duration (from a few 100 ms to up to 2.5 s), with these two parameters being directly correlated (Avoli and de Curtis, 2011). Hypersynchronous GABA_A_R-mediated potentials (pre-ictal spikes) have also been recorded in several areas of isolated guinea pig brain preparation during 4-AP application (Gnatkovsky et al., 2008; Uva et al., 2015). Our experiments and calculations revealed that IID1 is a purely GABA_A_R-mediated response; this explains why the slow interictal events induced by 4-AP are minimally affected by applying NMDAR antagonists and continue to occur in the presence of NMDA and non-NMDA glutamatergic receptor antagonists (Avoli and de Curtis, 2011).

Our observations were also consistent with recent *in vitro* and *in vivo* experiments showing that GABAergic interneurons are not merely responsible for providing inhibitory control of brain networks; rather, GABAergic inhibitory signals can favor seizure initiation (Gnatkovsky et al., 2008; Grasse et al., 2013; Shiri et al., 2016). Strong recruitment of interneurons and the consequent activation of postsynaptic GABA_A_Rs can lead to epileptiform synchronization via several mechanisms resulting from intracellular Cl^−^ accumulation, including 1) a positive shift in Cl^−^ reversal potential that makes GABA_A_R signaling excitatory and 2) an increase in local [K^+^]_o_ that is caused by KCC2 activity. In addition, it has been reported that excessive excitation of interneurons can result in their depolarization blocking and may synchronize neuronal populations through rebound excitation (Jirsa et al., 2014). Therefore, excessive activation of interneurons can be sufficient to disrupt the excitation/inhibition balance in the neuronal network, triggering ictal-like discharges (Avoli and de Curtis, 2011; Avoli et al., 2016).

The IID2s reported in the present study resemble some of the interictal events described in previous reports (Berretta et al., 2012; Hamidi et al., 2014; Herrington et al., 2015). However, due to the absence of whole-cell recordings in the studies cited, direct comparison of the events is difficult. In our preparation, IID2s were very close in properties to the clonic discharges observed during the late phases of SLEs (Avoli et al., 2002). In our preparation with low magnesium and high potassium solution containing 4-AP, IID2s usually followed the SLEs. The IID2s looked like building blocks of SLEs. Therefore, we suggest that high [K^+^]_o_ in combination with 4-AP and low [Mg^2+^]_o_ suppressed the inception of the tonic phase of SLEs and facilitated the emergence of IID2s. Indeed, in some slices, SLEs had only clonic ictal discharges, like the one in Figure 4A. In striking contrast to IID1s, IID2s depended on activation of both excitatory and inhibitory conductances. Our estimates showed a very pronounced, long-lasting NMDAR-dependent component of IID2s. The NMDAR-mediated conductance on the later stages of IID2 is much bigger than AMPAR-mediated conductance. The prolonged excessive activation of NMDARs with subsequent overload of Ca^2+^ entry is deleterious (Hardingham and Bading, 2010). Taking into consideration that synchronous hyperactivity of neurons could lead to excessive release of glutamate and its spillover, then activation of extrasynaptic NMDARs is expected. Recent studies have found that certain pro-death pathways or events are preferentially activated by extrasynaptic NMDARs compared to synaptic ones (Gouix et al., 2009; Xu et al., 2009; Hardingham and Bading, 2010). Thus, our results are in agreement with previous data that NMDARs may be responsible for the seizure-induced selective damage or excitotoxic cell death of certain neuronal populations as much as NMDAR antagonists provide protection against such damage (Meldrum, 1993; Parsons et al., 1999; Ghasemi and Schachter, 2011; Zaitsev et al., 2015).

The present study provided new data on synaptic conductances during IIDs, validated the data pharmacologically and verified their consistency with previous observations. Nevertheless, the mechanisms underlying the synaptic components themselves still are understood vaguely and need clarification by future studies that quantitatively match GABAR-mediated conductance to spiking activity of specific inhibitory cell subtypes as well as matñh glutamatergic conductances to firing of excitatory neurons. Our proposed method of estimating conductance and our measurements of the conductances during IIDs will aid future experiments and modeling studies of the mechanisms of seizure generation, development, and cessation.

## Author contributions

DA, AC, AZ designed the study. DA, JE performed experiments and analyzed data. DA, AC, JE, AZ made interpretation of data for the work. DA, AC, AZ wrote the manuscript. DA, AC, JE, AZ approved the final version.

## Funding

This work was supported by the Russian Science Foundation (project 16-15-10201).

### Conflict of interest statement

The authors declare that the research was conducted in the absence of any commercial or financial relationships that could be construed as a potential conflict of interest.
